# Endoscopic balloon dilation for the prevention of severe strictures caused by acute esophageal necrosis

**DOI:** 10.1002/deo2.43

**Published:** 2021-09-01

**Authors:** Ryuhei Jinushi, Naoki Ishii, Takahiko Yano, Noriatsu Imamura, Hisato Maekawa, Kenichi Kamachi

**Affiliations:** ^1^ Gastroenterology Center Tokyo Shinagawa Hospital Tokyo Japan

**Keywords:** acute esophageal necrosis, black esophagus, endoscopic balloon dilation

## Abstract

A 58‐year‐old man who had the history of alcohol dependence was referred to our emergency center due to severe nausea, vomiting, and subsequent onset of chest and back pain. Esophagogastroduodenoscopy (EGD) showed black‐appearing esophagus mucosa extending from the cervical esophagus to the esophagogastric junction with clear margins, a condition typically referred to as a black esophagus. Alcohol abuse was considered an important factor associated with acute esophageal necrosis in this patient. After admission, he received fluid resuscitation and proton‐pump inhibitors, with restriction of oral intake and treatment of alcohol dependence. Follow‐up EGDs and endoscopic balloon dilation were performed for the management of esophageal narrowing before the development of severe strictures. Strictures were successfully treated endoscopically without complications such as perforation.

## INTRODUCTION

Acute esophageal necrosis (AEN) is uncommon but one of important diseases encountered. AEN causes chest pain and/or upper gastrointestinal bleeding and presents as a black esophagus on esophagogastroduodenoscopy (EGD). A black esophagus is endoscopically characterized by diffuse circumferential black discoloration of the esophagus mucosa affecting the distal esophagus with a well‐defined demarcation at the esophagogastric junction (EGJ).^1^, ^2^ Severe stricture formation is an important complication of AEN, particularly during and/or after convalescence, and early intervention is essential to prevent and/or successfully treat severe strictures.^3^ If patients with AEN present with mediastinitis or esophageal perforation with abscess formation, surgical intervention is required. There are also reports of esophageal stenosis caused by AEN, including subtotal esophagectomy.^4^, ^5^ This report is a case of AEN associated with stricture formation in a patient who was successfully treated with endoscopic balloon dilation.

## CASE REPORT

**FIGURE 1:**
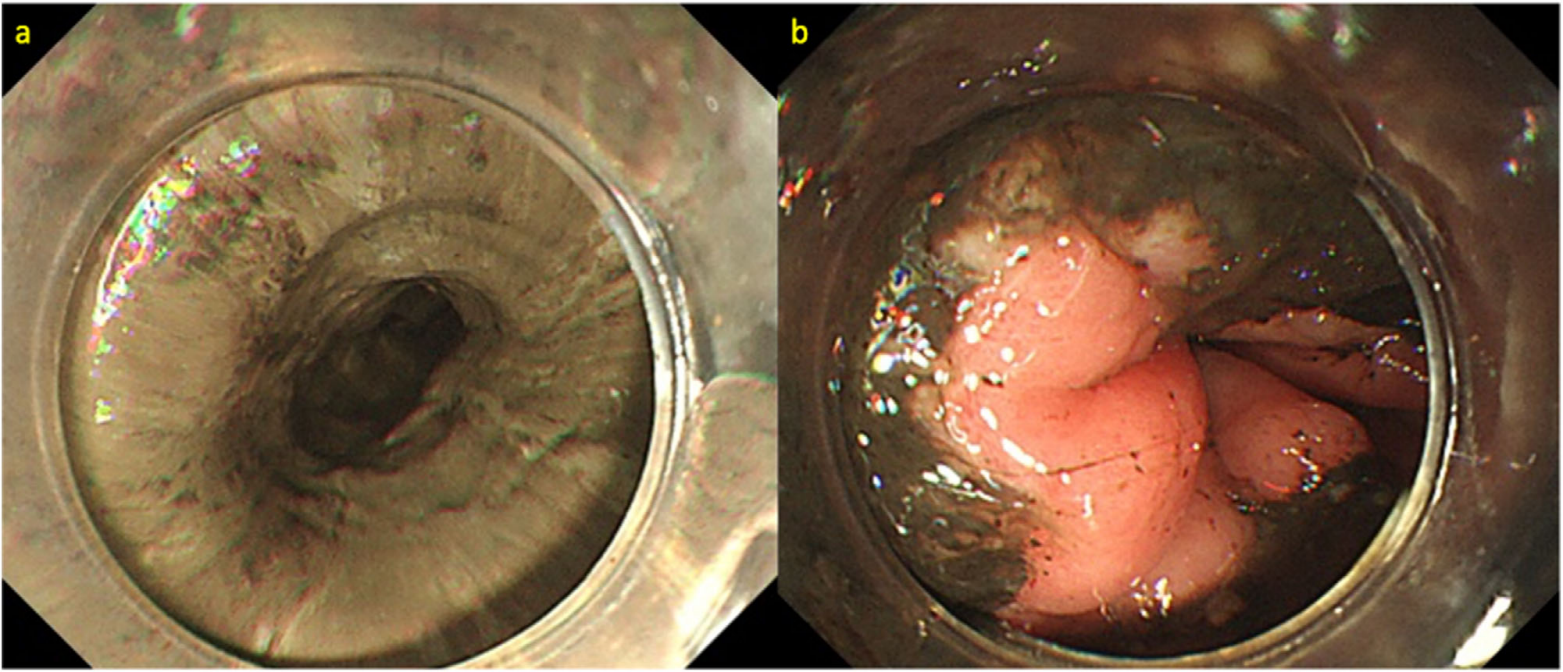
**(a and b)** EGD showed black‐appearing esophageal mucosa extending from the cervical esophagus to the EGJ. Circular muscle contraction was observed in the middle thoracic esophagus, with some residual muscle function throughout the same area

**FIGURE 2:**
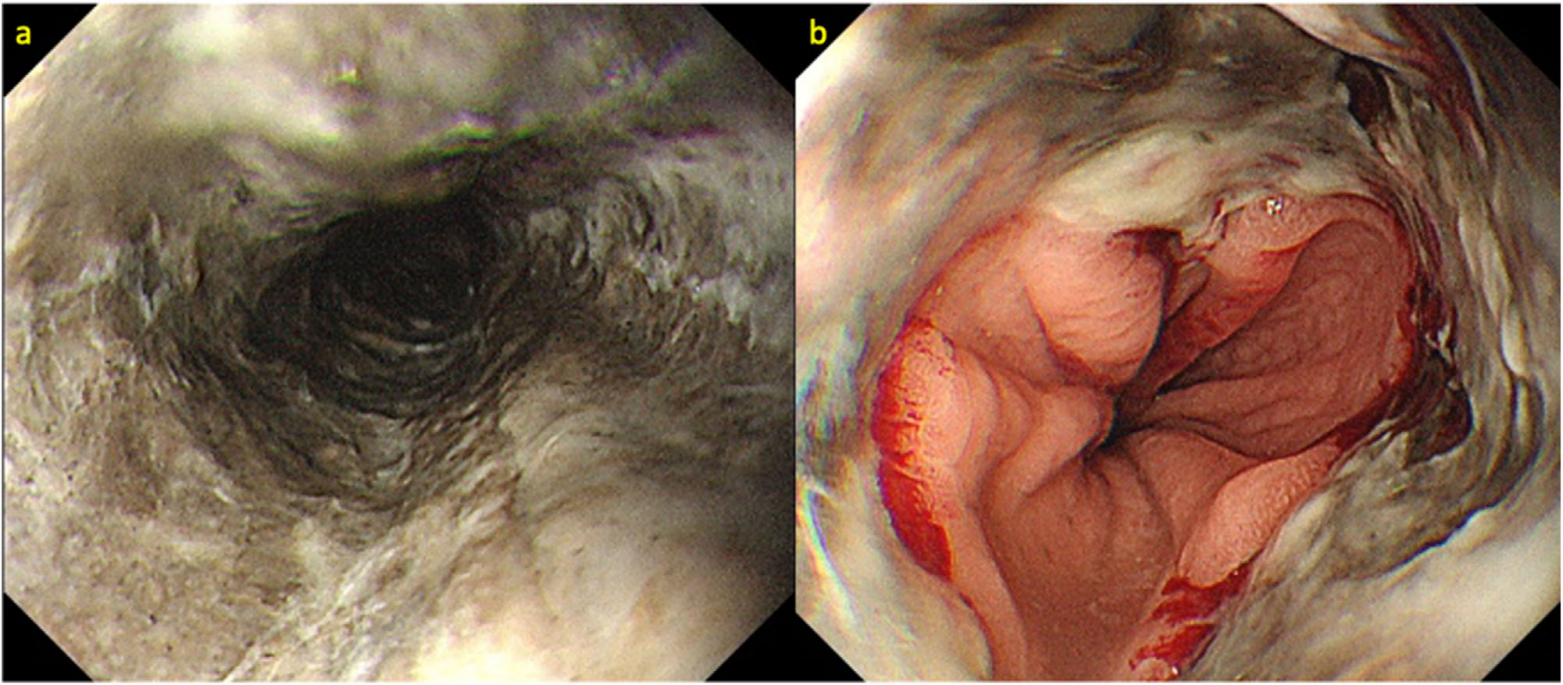
**(a and b)** Follow‐up EGD performed on the 6th day of hospitalization revealed that black discoloration was relieved, and perforation was not observed

**FIGURE 3:**
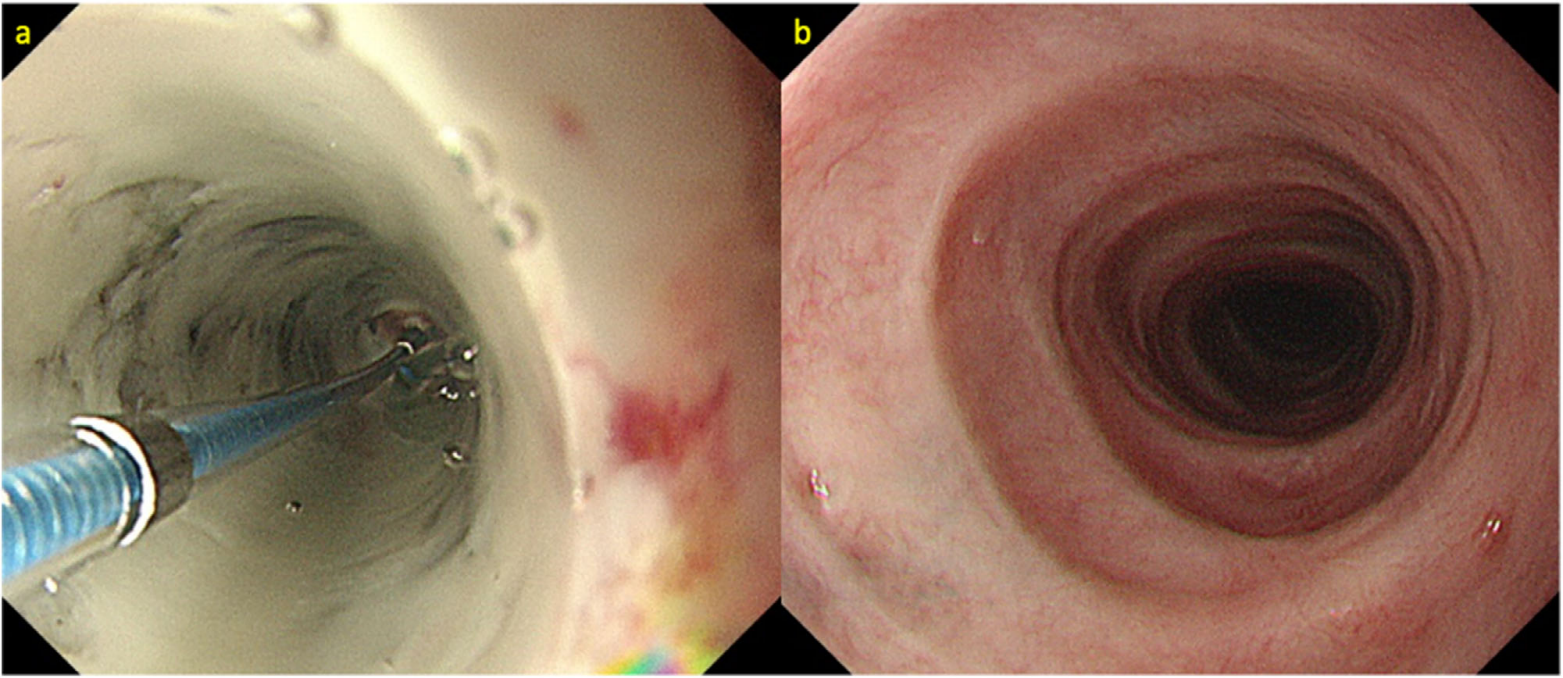
**(a)** Endoscopic balloon dilation was performed for the prevention of severe strictures. **(b)** Severe strictures were successfully prevented, and surgery could be obviated

A 58‐year‐old man diagnosed with alcohol dependence was referred to our hospital due to nausea, vomiting, and subsequent onset of chest and back pain. Physical examination showed no hypotension (blood pressure 124/76 mm Hg); however, we observed tachycardia (heart rate 118 beats/min) with pallor of the bulbar conjunctiva. Laboratory tests revealed lactic acidosis (pH [7.3], serum bicarbonate [18.5 mmol/L], anion gap [23.0 mmol/L], and serum lactic acid [4.0 mmol/L]) secondary to alcohol abuse. His serum hemoglobin level was 13.0 g/dl. He showed evidence of acute kidney injury (blood urea nitrogen 71.5 mg/dl, serum creatinine 5.1 mg/dl), elevated white blood cells (10.8 × 103/L), and increased serum C‐reactive protein (2.6 mg/dl) levels. Computed tomography (CT) was performed immediately upon admission to rule out life‐threatening conditions that could cause chest and back pain. CT revealed mild wall thickening along the entire length of the esophagus, with luminal fluid retention. We did not detect evidence of esophageal perforation and/or rupture or aortic dissection. EGD showed black‐appearing esophageal mucosa extending from the cervical esophagus to the EGJ. Circular muscle contraction was observed in the middle thoracic esophagus, with some residual muscle function throughout the same area (Figures 1a and 1b). We speculated the necrosis was confined to the submucosal layer. Treatment was aimed at restoration of hemodynamic stability via fluid resuscitation, intravenous administration of proton‐pump inhibitors (PPIs), and oral intake restriction, in addition to correction of underlying disorders such as alcohol dependence. Follow‐up EGD performed on the 6th day of the hospitalization revealed black discoloration of the esophagus that extended only for 20–40 cm from incisors; however, the black tone of the esophagus mucosa gradually faded and showed a tendency to improve (Figures 2a and 2b). Circular muscle contraction was clearly visualized. However, owing to progressive narrowing of the esophageal lumen, we performed follow‐up EGDs to treat any resultant strictures. EGD performed 4 weeks after hospitalization revealed esophageal stenosis in the middle thoracic esophagus; therefore, it was difficult to advance the scope (GIF‐H290). Esophageal balloon dilation (CRE; PRO GI Wireguided; Boston Scientific, Marlborough, MA, USA) was performed to treat stenosis, after which the scope could be advanced into the stomach (Figure 3a) . Subsequently, we observed improvement in the esophageal mucosa, as well as in his general condition, and the patient developed adequate insight into his alcohol abuse; therefore, we decided to perform follow‐up on an outpatient basis. The treatment strategy included oral PPI administration and continued follow‐up with weekly EGDs and balloon dilation. By the 15th week after discharge, scar formation was observed, although the scope could be advanced through the narrowed lumen into the stomach without esophageal balloon dilation (Figure 3b). No aggravation or recurrent symptoms occurred during the follow‐up period of 8 months. A total of 21 EGDs, 15 of which required esophageal balloon dilation, were performed.

## DISCUSSION

AEN can cause a wide variety of severe symptoms, including chest pain and gastrointestinal bleeding, and can result in life‐threatening complications. Perforations are rarely seen but often fatal complication of AEN, and clinicians should be conscious of this possibility.^3^ CT was done for the evaluation of severe chest pain on admission and was actually important to exclude catastrophic conditions such as esophageal perforation and/or rupture and aortic dissection in our patient. After that, EGD could be performed for further evaluations. The most serious complication of AEN is perforation, which is reported to occur in about 7% of patients, and the sequelae are stenosis formation, which occurs in more than 10% of patients.^3^ Reportedly, alcohol abuse, atherosclerosis, hypertension, diabetes mellitus, dyslipidemia, cardiovascular and renal diseases, sepsis, trauma, hypoalbuminemia, ischemic insult, and a compromised mucosal defense mechanism can serve as important risk factors for AEN.^1^, ^2^, ^3^, ^6^ The high mortality rate is primarily associated with exacerbation of comorbidities in patients with AEN; certainly, some studies have reported mortality rates > 30% in the absence of appropriate treatment.^2^, ^3^, ^6^ Although no specific treatment is established for AEN, early diagnosis, improvement of patients’ deteriorated health conditions, treatment of comorbidities, drug therapies including PPI administration, and restoration of hemodynamic stability are important for effective managements.^6^ Our patient developed AEN secondary to alcohol abuse and was referred on unstable vital signs. However, the patient could be successfully treated with bowel rest, sufficient fluid resuscitation, and PPI administration in addition to effective communication and patient education to ensure that he developed clinical insight into his devastated condition due to alcohol dependence. Although most AEN cases usually improve with follow‐up, some patients with AEN develop severe strictures during the convalescence phase, as in this case, and may require surgical interventions. Therefore, we performed follow‐up EGDs and endoscopic balloon dilation for the narrowed esophagus to prevent the development of severe strictures in our patient. Early endoscopic balloon dilation effectively obviated surgery and maintained the patient's quality of life in this case.

## CONFLICT OF INTEREST

The authors declare that there is no conflict of interest that could be perceived as prejudicing the impartiality of the research reported.

## FUNDING INFORMATION

None.

## References

[1] Siddiqi A , Chaudhary FS , Naqvi HA , Saleh N , Farooqi R , Yousaf MN . Black esophagus: A syndrome of acute esophageal necrosis associated with active alcohol drinking. BMJ Open Gastroenterol 2020; 7: e000466. 10.1136/bmjgast-2020-000466 PMC7422689 32788199

[2] Gurvits GE , Cherian K , Shami MN , *et al*. Black esophagus: New insights and multicenter international experience in 2014. Dig Dis Sci 2015; 60: 444–53. 25297468 10.1007/s10620-014-3382-1

[3] Gurvits GE . Black esophagus: Acute esophageal necrosis syndrome. World J Gastroenterol 2010; 16: 3219–25. 20614476 10.3748/wjg.v16.i26.3219 PMC2900712

[4] Goldenberg SP , Wain SL , Marignani P . Acute necrotizing esophagitis. Gastroenterology 1990; 98: 493–6. 2295407 10.1016/0016-5085(90)90844-q

[5] Kim YH , Choi SY . Black esophagus with concomitant candidiasis developed after diabetic ketoacidosis. World J Gastroenterol 2007; 13: 5662–3. 17948944 10.3748/wjg.v13.i42.5662 PMC4172749

[6] Uyar S , Duman A , Tan A , *et al*. Black esophagus. Turk J Gastroenterol 2019; 30: 986–7. 31767554 10.5152/tjg.2019.181019 PMC6883997

